# Sleep duration and risk of obesity among a sample of Victorian school children

**DOI:** 10.1186/s12889-016-2913-4

**Published:** 2016-03-09

**Authors:** Bridget Morrissey, Mary Malakellis, Jill Whelan, Lynne Millar, Boyd Swinburn, Steven Allender, Claudia Strugnell

**Affiliations:** School of Health and Social Development, Deakin University, 221 Burwood Hwy, Burwood, Victoria 3125 Australia; World Health Organization’s Collaborating Centre for Obesity Prevention, Deakin Population Health, Deakin University, Geelong, Australia; The CO-OPS Collaboration, WHO-CC, Deakin Population Health, Deakin University, Geelong, Australia; School of Population Health, The University of Auckland, Auckland, New Zealand

**Keywords:** Sleep, Sleep duration, Children, School children, Overweight, Obesity, Accelerometry, ActiGraph, Physical activity, Sedentary behaviour

## Abstract

**Background:**

Insufficient sleep is potentially an important modifiable risk factor for obesity and poor physical activity and sedentary behaviours among children. However, inconsistencies across studies highlight the need for more objective measures. This paper examines the relationship between sleep duration and objectively measured physical activity, sedentary time and weight status, among a sample of Victorian Primary School children.

**Methods:**

A sub-sample of 298 grades four (n = 157) and six (n = 132) Victorian primary school children (aged 9.2-13.2 years) with complete accelerometry and anthropometry data, from 39 schools, were taken from a pilot study of a larger state based cluster randomized control trial in 2013. Data comprised: researcher measured height and weight; accelerometry derived physical activity and sedentary time; and self-reported sleep duration and hypothesised confounding factors (e.g. age, gender and environmental factors).

**Results:**

Compared with sufficient sleepers (67 %), those with insufficient sleep (<10 hrs/day) were significantly more likely to be overweight (OR 1.97, 95 % CI:1.11-3.48) or obese (OR 2.43, 95 % CI:1.26-4.71). No association between sleep and objectively measured physical activity levels or sedentary time was found.

**Conclusion:**

The strong positive relationship between weight status and sleep deprivation merits further research though PA and sedentary time do not seem to be involved in the relationship. Strategies to improve sleep duration may help obesity prevention initiatives in the future.

## Background

The underlying determinants of childhood overweight and obesity have been the subject of much research globally [1, 2] and it has been established that physical activity (PA), sedentary time (ST) and dietary intake are key modifiable risk factors [2–5]. Recent thinking suggests a widening of the range of potential modifiable factors to consider the association of insufficient sleep [2, 6, 7]. Findings from recent studies indicate that children who sleep for insufficient durations (<10 hrs) are more likely to exhibit higher Body Mass Index (BMI), waist circumference (WC) and obesity rates [8–10].

While some variation in recommendations exist, it is commonly accepted that children aged five to 12 years receive between 9 and 11 hours of sleep per night [11, 12]. Internationally average sleep durations have been decreasing over recent decades [13]. Australian data has reported a decline in average sleep durations of approximately 28 mins and 33 mins (for girls and boys respectively) among Australian children between 1985 and 2004 [14]. The global trend in decreasing sleep has occurred at the same time as increases in the prevalence of overweight and obesity [13–15]. These trends are supported by studies that found insufficient sleep among children was associated with an increased risk of overweight and obesity [16–19], reduced physical activity and increased sedentary behaviours [9, 16, 17]. Children’s increased screen time and use of electronic devices around bedtime has also been theorised as a potential link to these trends [19]. 

A reoccurring issue of available literature on children’s sleep, weight status PA and SB is the predominant utilisation of subjective measures [14, 17]. While some studies have incorporated objective measures, some findings are conflicting and inconsistencies in measurement methods make comparison of results difficult [20]. Findings from two studies utilising wrist-worn accelerometers [21, 22] and one using hip-worn accelerometry [23] to objectively measure children’s activity have contrasted greatly. Gupta et al. (2002) reported that poor sleep over a 24 hour period lead to significant reductions in next day PA [22], while Eskedt et al. (2013) reported no significant association between sleep duration and PA levels over a 7 day period [21]. Contradicting both of these studies, hip-worn accelerometry data from Nixon et al. (2008) found that higher levels of daytime activity were associated with reduced sleep durations [23]. Factors such as not separating children from adolescents (as sleep needs change from childhood to adolescence) may have influenced results among the sample of children aged 11–16 years old in Gupta et al. [22]. The restricted length of monitoring in this study (24 hrs) may also not be fully representative of usual behaviour [24]. On top of these, the wear-site of accelerometers has been reported to influence readings, with a validation study reporting that wrist accelerometry data tends to significantly over estimate children’s PA and underestimate ST [20].

As Australian studies investigating the relationship between children’s sleep, weight status PA and SB have been cross-sectional in nature and reliant on subjective measures [17, 25–27], the use of hip worn accelerometry to objectively measure children’s PA and SB would strengthen our understanding of the association between sleep and these factors among Australian children [17, 25–27].

In this study we determine the relationship between PA, ST and weight status on sleep duration, among a sample of Victorian Primary School children, using objective measures of PA and ST.

In this paper it is hypothesised that, compared with sufficient sleepers, children with insufficient sleep durations will more likely be overweight or obese and record lower average daily PA and higher average daily ST. It is also hypothesised that environmental factors around increased screen behaviours (the number of TVs per house and having a TV or electronic gaming device in the bedroom) will significantly reduce children’s sleep duration.

## Methods

This study utilised a sub-sample of cross-sectional data on grades four and six primary school children from a pilot study (2013), of a state-based cluster randomised control trial, the methods of which have previously been published (Strugnell et al., under review).

A random sample of 156 primary schools were invited to participate, of which 39 consented (school-level response rate (RR) = 25 %). All grades four and six students within these schools were then invited to participate and provided with plain language statements and consent forms. This pilot study used opt-in consent, where participation required a parent/guardian signed consent form. Of the 2,357 invited, 839 students returned the consent form, enabling their participation (student-level RR = 35.6 %). In order to analyse objectively measured PA and ST, only data from a sub-sample of students who were provided with an accelerometer (N = 373) was used.

### Measures

#### Anthropometric measures

Trained data collectors collected measurements on children’s height (HM200P Stadiometer, Charder Electronic Co, Ltd), weight (UC-321 scale, A&D Australasia Pty Ltd) and waist circumference (WC) (Lufkin W606PM metal tape measure, Apex Tool Group, LLC), following the procedure previously published [28]. Children’s measurements were taken wearing one layer of light clothing (e.g. t-shirt and pants) with shoes and jumpers removed. All measurements were taken twice, to the nearest 0.1 cm for height and waist and the nearest 0.01 kg for weight, with a third measurement required for any discrepancies greater than 0.5 cm or 0.5 kg. The mean height and weight measurements were used to generate BMI-z scores according to the WHO international BMI growth reference standards [29].

#### Sleep duration

Children were asked “During the past 7 days, how much time did you usually spend sleeping per night?” and then selected one of the nine options (“less than 5 hours”, “5 hours” “6 hours”, “7 hours”, “8 hours”, “9 hours” “10 hours”, “11 hours”, or “more than 12 hours”). Aligning with recommendations by the Australian Sleep Health Foundation [12] and classifications used in previous literature [17, 30, 31], sufficient sleep was categorised as 10 or more hours of sleep per night and less than this as insufficient sleep.

#### Physical activity and sedentary time

Every second student across genders and grade levels (e.g. 1^st^, 3^rd^, 5^th^ for boys and girls in Grade 6 etc.) were invited to wear a waist-worn ActiGraph GT3X or GT3X+ accelerometer (ActiGraph, Pensacola, FL) to collect objectively measured data on daily PA and ST. Participants were instructed to wear the monitor on the right hip during waking hours for seven days, except during water-based or high contact activities (e.g. martial arts).

To reduce the possibility for any discrepancies between ActiGraph models they were both programed with a 15-second epoch and a 30-hertz sampling rate [32]. A valid day was considered if ≥600 minutes per day (mins.d^−1^) of wear time was recorded, according to the criteria of wear/non-wear time by Troiano and colleagues (2008) [33], over a minimum of three days. Average daily moderate to vigorous physical activity (MVPA), light intensity physical activity (LPA) and ST were determined by summing the time spent in each category according to 3-axial activity count cut-points developed for children aged 10–15 years [34]. Using the average day method outlined by Olds (2007) [35] total average MVPA time was dichotomised according to the Australian PA guidelines for children, into an average of ≥60 min/day (guidelines met) or an average of <60 min/day (guidelines not met) [36, 37], allowing for categorical analysis of children’s PA. As there are currently no specific daily ST guidelines (except for the restriction of screen time to 2 hrs/day) or daily LPA [38, 39], low, moderate and high tertiles were created to enable categorical comparison of these variables with sleep duration.

#### Demographic variables

Demographic items included age, gender and whether English was the only language spoken at home.

#### Confounding variables

Participants were asked to report on environmental factors surrounding sedentary behaviours, the presence of a TV in the bedroom (Yes/No) as well as having an electronic gaming device (EGD) or laptop/computer in the bedroom (Yes/No). Clustering by Local Government Areas was included to account for the sampling design.

### Statistical analysis

STATA SE12 (Stata Corporation, Texas, USA) was used for all statistical analysis. Aligned with recommendations from previous studies [16, 24, 40–41], only participants with ≥3 days of accelerometry wear-time data were included for analysis (N = 298; 80 % of possible participants). Parametric tests for normality were conducted on all independent variables, resulting in participants with values ±3SD from the mean on independent variables of MVPA, LPA, ST, BMI, and WC being omitted from the relevant analysis (n = 9).

Chi-squared and paired t-tests were used to assess gender differences for each variable. Chi-square tests were conducted to assess potential differences between insufficient and sufficient sleep duration and weight status, LPA, MVPA and ST, using the categorical expression of each variable. Lastly, multivariate hierarchical logistic regression analyses were used to calculate the effect of sleep duration on weight status after adjusting for hypothesised covariates including age, gender, Socio-Economic Indexes for Areas (SEIFA), PA, ST, TV in bedroom and computer/EGD in the bedroom.

## Results

Of the 289 participants (56 % girls; 54.3 % grade four) with valid accelerometer data 30.5 % were categorised as overweight or obese (36.2 % boys, 25.9 % girls), 87 % came from English speaking homes and 52.5 % lived in areas categorised as being in the lower two SEIFA quintiles (Table 1).

**Table 1:** Unadjusted descriptive statistics^§^

		All (N=289)	Boys (N=127)	Girls (N=162)
Age (years)		11.2 ± 1.0	11.3 ± 1.1	11.1 ± 1.0
English sole language at home (% ) ^a^		87.0	86.9	87.0
SEIFA (%)*	1^st^ Quintile	24.6	31.1	19.8
	2^nd^ Quintile	27.9	27.7	28.0
	3^rd^ Quintile	12.0	8.4	14.7
	4^th^ Quintile	30.4	28.6	31.9
	5^th^ Quintile	5.1	4.2	5.7
BMI-z *		0.4 ± 1.1	0.6 ± 1.2	0.3 ± 1.1*
Overweight/Obese (% ) ^b^*		30.5	36.2	25.9*
WC (cm) **		66.1 ± 8.7	67.6 ± 9.3	64.9 ± 8.1**
Average no of accelerometer days		5.1 ± 1.3	5.2 ± 1.3	5.1 ± 1.3
Average wear time (min.day-1)		786.1 ± 70.1	784.7 ±73.1	787.1 ±67.9
MVPA (min.day-1) **		101.0 ± 31.0	116.5 ± 29.2	88.9 ± 26.7**
Precent meeting PA Guidelines (%) **		91.4	96.9	87.0**
LPA (min.day-1)**	Total LPA	196.9 ± 33.7	191.4±28.8	201.1 ± 36.6**
	Low LPA	160.3±16.3	163.9±13.0	155.9±18.9
	Med LPA	196.3±8.2	196.6±8.4	196.1±8.0
	High LPA	233.9±19.2	220.1±12.5	236.4±21.6
ST (min.day-1) *	Total ST	487.2 ± 81.2	475.9±83.4	496.1±78.6*
	Low ST	406.8±36.9	396.2±40.8	416.7±29.8
	Med ST	481.9±15.3	480.6±16.3	482.9±14.6
	High ST	578.4±59.5	574.1±58.7	581.3±60.4
Sleep Duration (%)	<5	2.1	3.1	1.2
	5	1.0	0.8	1.2
	6	2.8	3.9	1.9
	7	3.1	3.1	3.1
	8	7.3	7.9	6.8
	9	17.0	15.0	18.5
	10	38.0	41.7	35.2
	11	18.7	12.6	23.5
	≥12	10.0	11.8	8.6
Sleep Category (%)				
Insufficient (<10hrs/night)		33.2	33.9	32.7
Sufficient (≥10hrs/night)		66.8	66.1	67.3
TV in bedroom (%) ^a^		31.8	33.1	30.9
Number of TVs in house (%)	<2	40.8	36.2	44.4
	3-4	40.5	43.3	38.3
	≥5	18.7	20.5	17.3
Computer/Elec. games in bedroom(%) ^a^ *		39.3	45.2	34.8*

§ ±3SD on BMI, waist, ST, MVPA and LPA excluded

^a^ Divergent number of participants: English sole language at home: N=122 for boys; TV in bedroom: N=124 for boys; Computer/Elec. Games in bedroom: N=124 for boys and N=161 for girls

^b^ defined by WHO World Health Organization. (1)

* p<0.05 for sex difference; ** p<0.01 for sex difference

SEIFA - Socio-Economic Indexes for Areas; WC–waist circumference; MVPA-moderate to vigorous physical activity; LPA-low intensity physical activity; ST- sedentary time

1. World Health Organization. WHO Child Growth Standards: Length/height-for-age, weight-for-age, weight-for-length, weight-for-height and body mass index-for-age, Methods and development. 2006

A third (33.2 %) of all participants were classified as receiving insufficient sleep, with no significant gender differences (x^2^ = 9.68, p = 0.84). Of those categorised as insufficient sleepers, 39.6 % were also classified as either overweight (25.0 %) or obese (14.6 %). This proportion was significantly greater than the proportion of sufficient sleepers categorised as overweight or obese (25.9 %; x^2^ = 5.66, p = 0.02).

No significant difference was found (x^2^ = 3.46, p = 0.12; and x^2^ = 2.21, p = 0.33, respectively) between the proportion of children categorised as overweight or obese with or without accelerometry data, or between participants with valid or invalid accelerometry wear-time. However, sleep categorisation was found to differ significantly between the excluded participants and analysed sample (x^2^ = 9.72, p = 0.00), with the current sample displaying an under-representation of overweight/obese insufficient sleeper (13 %) compared to non-analysed (16 %).

A multivariate hierarchical logistic regression (Model 1; Table 2) shows that insufficient sleepers were more likely to be in the overweight range (OR 1.72; 95 % CI:1.10-2.68) compared to the normal weight range but no such association was observed for those in the obese range (OR 1.80; 95 % CI:0.94-3.45). Adjustment of the initial model for age, gender, SEIFA and study condition (Model 2; Table 2) shows insufficient sleepers more likely to be categorised as overweight (OR 1.88; 95 % CI:1.14-3.13) or obese (OR 2.31; 95 % CI:1.18-4.53). Of the demographic variables only age was related to sleep duration (OR 1.59; 95 % CI:1.17-2.16:). Model 3 shows that after further adjustment for environmental characteristics the relationship between weight status and sleep remains significant (overweight OR 1.97; 95 % CI:1.11-3.48 and obese OR 2.43; 95 % CI:1.26-4.71) as does age (OR 1.57; 95 % CI:1.15-2.13). Presence of a computer/EGD in the bedroom was related to sleep duration and insufficient sleepers were twice as likely to have one (or more) of these devices in the bedroom. (OR 2.11; 95 % CI:1.25-3.59). Across all three models no significant association was found between children’s sleep and their MVPA, LPA or ST.

**Table 2:** Multiple logistic regression: with odds ratios according to sleep category (sufficient vs. insufficient)

Variable	Model 1		Model 2		Model 3	
	OR	95 % CI	OR	95 % CI	OR	95 % CI
Weight Status						
Overweight	1.720*	1.103-2.681	1.888*	1.138-3.131	1.968*	1.112-3.481
Obese	1.800	0.940-3.446	2.306*	1.175-4.526	2.433**	1.256-4.711
Activity Levels						
MVPA	1.002	0.991-1.014	1.003	0.990-1.016	1.003	0.991-1.015
LPA	0.999	0.990-1.009	1.001	0.992-1.010	1.001	0.992-1.010
ST	1.003	0.999-1.006	1.002	0.999-1.005	1.002	0.999-1.004
Demographic						
Age (Years)			1.593**	1.174-2.162	1.565**	1.150-2.128
Gender			1.192	0.593-2.396	1.276	0.666-2.442
SEIFA			1.036	0.854-1.256	1.059	0.880-1.275
Condition			0.829	0.460-1.493	0.848	0.465-1.546
Environmental						
TV Room					1.100	0.574-2.105
Comp Room					2.115**	1.245-3.593

Model 1: Unadjusted logistical regression on hypothesised correlated variables; Model 2:Logistical regression, adjusted for demographic covariates (Age, Gender & SEIFA) and condition; Model 3: Logistical regression, further adjusted for environmental covariates (TV in room & Computer/EGD in bedroom)

OR – odds ratio; CI – confidence interval; *p<0.05; **p<0.01;

## Discussion

The hypothesis that children with insufficient sleep are more likely to be categorised as being overweight or obese, less physically active and more sedentary was partly supported. Using objective measures of height and weight we found that weight status was inversely associated with children’s self-reported sleep duration. In contrast to previous studies no relationship was observed between PA, ST and sleep duration. However, in the current study age and presence of computer/EGD in the bedroom were inversely associated with children’s sleep duration.

In this study, a third of participants were categorised as insufficient sleepers. The high levels of sleep deprivation among the current sample is comparable with that reported by Shi et al. (2010), where 48.2 % of their sample of 3,495 South Australian children (5–15 years) were reported sleeping less than 10 hours per night by their parents [17]. Other estimates of insufficient sleep are higher; a nationally representative sample of 6,324 Australian 7–15 year olds found that almost two-thirds (62.4 %) reported not getting the recommended 10+ hours of sleep per night [26]. This may suggest that the national prevalence of sleep deprivation among children could be an even more substantial issue outside of the two states of South Australia and Victoria, or most likely be due to the difference in self-report versus parent-report measures used.

Despite these disparities, the findings about the size and direction for the relationship between sleep and children’s weight status found in this study are consistent among local and international studies such as those by Shi et al. (2010) and Seegers et al. (2011) [17, 42]. Our results suggest that children reporting insufficient sleep were almost twice as likely to be overweight and almost two and a half times more likely to be obese than those meeting sleep duration recommendations. Our study supports international findings from 1,916 10–13 year olds in Quebec showing insufficient or shorter sleep durations among children increased the odds of being obese by 1.41 (95 % CI:1.24, 1.61) times [42].

As several national sleep guidelines have been extended recently, suggesting nine and potentially eight hours of sleep per night might be sufficient for some children [12, 43], additional analyses were conducted to explore how adjusting the categorization of insufficient sleep influences results among the current sample. While our initial analysis indicated sleeping ≤9 hours per night was shown to increase the odds of overweight and obesity compared with sleeping ≥10 hours, when re-categorising insufficient sleep as ≤8 or ≤7 hours of sleep per night no significant association was found. However, the reduced power due to the low representation of participants in these categories (only 16 % sleeping ≤8 hours per night and only 9 % sleeping ≤7 hours) makes it difficult to assume that these cut-points would not reach significance among larger samples. This highlights the importance to consider the cut-points being used to determine sufficient sleep in future studies, as well as the need for larger more representative samples.

We found no relationship between children’s sleep duration and average MVPA, LPA and ST, which contrast findings from previous studies [9, 16, 17, 21]. The lack of association between these factors in the current study could be due to a number of reasons including: the differentiation between previous subjectively measured PA and ST compared with the current objective accelerometry data; the slightly smaller sample size due to the restriction of available accelerometers; or due to characteristics surrounding the type of participants who adhered to wear-time requirements and therefore were included in the analysis.

There has been some indication that participants with higher BMI, waist circumference and sedentary behaviours may be less likely to meet wear-time requirements [44]. In such cases, results may not fully represent the study population and may have produced an overestimation of the sample population’s PA and underrepresentation of average ST [44]. However, our analysis does not support this suggesting no significant weight status differences between children with valid versus invalid accelerometry wear-time, or between participants with or without accelerometry data.

Although we found no significant association between sleep duration and children’s ST in the current study, it is interesting to note that having a computer/EGD in the bedroom was associated with children’s sleep durations. Children who reported having a computer/gaming console in the bedroom had twice the odds for not receiving the recommended ≥10 hours of sleep per night. One potential explanation for this relationship suggests that sedentary behaviours involving computer/EGD directly deduct from children’s sleep duration by interfering with time that should be dedicated to sleeping, but not necessarily influencing their overall average daily ST [45]. It has also been suggested that exposure to artificial light created by these screen behaviours may lead to disruptions in the circadian rhythm, with an increase in alertness and decreased sleep onset and duration [46]. It may be useful for future research to examine the times of day that children engage in screen based activities so that recommendations can be extended to include guidelines around screen-free times in order to promote sufficient sleep.

Another possible explanation for the association between computers/EGDs and insufficient sleep among children may be linked within the significant inverse association between children’s sleep and their age, demonstrated in the current and previous studies [17, 21, 47]. While literature suggests this age associated decline in sleep duration could be related to later bed times of older children without adjusting wake-up times [14, 48], it has been proposed that it might be due the higher usage of electronic devices (such as computers, phones and TVs) in older children/adolescents [9, 21, 47–49].

More work is need to gain a deeper understanding on the modifiers and confounders of the association between children’s sleep and weight status. Despite not finding an association between objectively measured average daily PA and ST, our findings of an association between electronic devices such as computers/EGD and reduced sleep duration highlights the need to better understand how these behaviours influence children’s sleep. Future studies would also benefit from more stringent measures on sleep duration (such as accelerometers).

## Conclusion

Among this sample of Victorian primary school children sleep duration was inversely associated with weight status, though not between objectively measured PA and ST. Insufficient sleep was significantly higher among children with a computer/EGD in their bedroom. The findings suggest sleep is a plausible target behaviour for obesity prevention initiatives.

## Ethics

Ethics approval was received from the Deakin University’s Human Research Ethics Committee, the Victorian Department and Early Childhood Development and the Catholic Education Office Archdiocese’s of Melbourne, Sale, Sandhurst and Ballarat for this study.

## Abbreviations

PA: physical activity
ST: sedentary time
BMI: Body Mass Index
WC: waist circumference
LPA: light intensity physical activity
MVPA: moderate to vigorous physical activity
EGD: electronic gaming device
SEIFA: Socio-Economic Indexes for Areas
